# Draft genome sequence of *Candida auris* MUTCD0001: the first clinical isolate from Ireland

**DOI:** 10.1128/mra.00178-25

**Published:** 2025-05-28

**Authors:** Kayleigh Sines, Matthew Amoo, Joy Clarke, Kevin Kavanagh, Eddie McCullagh, Julie Renwick, David A. Fitzpatrick

**Affiliations:** 1 Department of Biology, Maynooth University 98865, Maynooth, County Kildare, Ireland; 2 Mercy University Hospital, Grenville Place, Cork, Ireland; 3 Department of Clinical Microbiology, School of Medicine, Trinity College Dublin, Trinity Centre for Health Science, Tallaght University Hospital 57976, Dublin, Ireland; University of California Riverside, Riverside, California, USA

**Keywords:** *Candida auris*, genome, multidrug resistance, clinical isolate, Ireland

## Abstract

Here, we report the draft genome sequence of *Candida auris* MUTCD0001, the first isolate of a human clinical specimen from Ireland. *C. auris* is a multidrug-resistant yeast, and studies of drug-resistant *C. auris* outbreaks have shown transmission among patients, demonstrating a need for improved epidemiological surveillance of *C. auris*.

## ANNOUNCEMENT

*Candida auris* is a multidrug-resistant yeast and was recently added to the WHO’s Fungal Priority Pathogen List (1). High rates of multidrug resistance have contributed to significant mortality among hospitalized patients suffering from invasive *C. auris* infections (2). In Ireland, four cases of *C. auris* have been reported since it became notifiable in 2017. Here, we report the draft genome sequence of the first clinical isolate from Ireland.

In August 2022, *C. auris* was isolated and cultured from the abdominal tissue of a patient from the High Dependence Unit of Cork University Hospital, Ireland (global positioning system coordinates 51.88 N, 8.51 W) and initially identified by Brucker matrix-assisted laser desorption ionization-time of flight (MALDI-TOF) analysis. Liquid cultures in YEPD (2% glucose, 2% peptone, and 1% yeast extract) were prepared by inoculation with a single colony from isolate YEPD plate cultures. Liquid cultures were grown overnight at 37°C at 200 rpm. Total genomic DNA was extracted from the stationary phase using the Quick-DNA Fungal/Bacterial Miniprep Kit (Zymo Research). DNA concentration and quality were assessed using a Nanodrop 2000 Spectrophotometer (ThermoFisher Scientific). Sequencing libraries were constructed using the Illumina TruSeq DNA Library Prep Kit. The genome was sequenced by 150 bp paired-end sequencing on an Illumina NovaSeq 6000 system using Novogene.

In total, 5,324,570 paired-end reads were obtained. Low-quality reads (Q scores < 20) were removed using Skewer v0.2.2 (3), and high-quality reads (5,324,513) were assembled using SPAdes v3.13.0 (4) with default parameters. The resultant assembly consisted of 112 contigs. The total size of the genome was 12,314,949 bp, the N50 value was 216,007 bp, and the G + C content was 44.52%. The largest contig in the assembly was 514,360 bp. Assembly completeness was assessed at 99.2% using BUSCO v5.8.0 with the saccharomycetes_odb10 data set (5). Species identification was further confirmed using the internal transcribed spacer sequence, which was found to be 100% identical to *C. auris* (NCBI accession number HE797772.1).

Single-nucleotide polymorphisms (SNPs) were called using GATK v4.3.0.0 (6) against representative genomes from the five established *C. auris* clades, namely strains B8441 (clade I), B11220 (clade II), B11221 (clade III), B11245 (Clade IV), and B18474 (Clade V). Reads were aligned to representative genomes using BWA-MEM ver. 0.7.17 (7). Aligned BAM files were sorted, and duplicate reads were marked using GATK SortSam and MarkDuplicates tools, respectively. Variants were called with the GATK HaplotypeCaller using the default settings and filtered using GATK VariantFiltration with the following filter flags: “QD <2.0,” “FS >60.0,” “MQ <40.0,” “SOR > 4.0,” “MQRankSum <−12.5,” “ReadPosRankSum <−8.0.” GATK SelectVariants was used to extract filtered SNPs. The MUTCD0001 isolate is most closely related to B8441, with 1,242 SNPs, which identifies it as a clade I isolate (Table 1). The complete genome sequence also contains clade I-specific primers (8). Gene mutations associated with antifungal resistance, including mutations to *ERG11,* were observed (9).

**TABLE 1:** Number of SNPs identified in *Candida auris* MUTCD0001 relative to clade reference genomes

Clade	Reference genome	Total #SNPS	GenBank accession	Citation
I	B8441	1,242	GCA_002759435.3	(10)
II	B11220	66,810	GCA_003013715.2	(11)
III	B11221	11,636	GCA_002775015.1	(10)
IV	B11245	44,540	GCA_008275145.1	(12)
V	B18474	275,640	GCA_016809505.1	(12)

Genome surveillance is implemented by national public health agencies. Moving forward, genomic monitoring in Irish hospitals and long-term care facilities should be considered.

## Data Availability

The Bioproject ID is PRJNA1244779. The BioSample ID is SAMN47730786. The raw Illumina reads are available at ENA/SRA under the accession number SRR32934938. This Whole-Genome Shotgun project has been deposited at DDBJ/ENA/GenBank under the accession JBMWUU000000000. VCF files have been deposited as an archive to Zenodo (DOI:10.5281/zenodo.15119972).
